# Explainable AI-enabled adaptive fuzzy MPPT and energy management for bifacial PV and battery-powered electric vehicle charging system

**DOI:** 10.1038/s41598-025-34894-4

**Published:** 2026-01-07

**Authors:** Vineet Kumar Tiwari, Awadhesh Kumar, Shekhar Yadav, Dinesh Kumar Nishad, Saifullah Khalid

**Affiliations:** 1 Department of Electrical Engineering, Madan Mohan Malaviya University of Technology, Gorakhpur, India; 2 Department of Electrical Engineering, Dr. Shakuntala Misra National Rehabilitation University, Lucknow, India; 3 IBMM research, Khartoum, Sudan

**Keywords:** Adaptive fuzzy logic, Explainable artificial intelligence, Maximum power point tracking, Bifacial solar PV, Electric vehicle charging, Energy management system, Energy science and technology, Engineering

## Abstract

The widespread adoption of electric vehicles (EVs) requires efficient, sustainable charging infrastructure. The use of bifacial photovoltaic (PV) panels with battery energy storage systems (BESS) will provide uninterrupted, sustainable power to EV charging stations. This research is a combination of both an Explainable Artificial Intelligence (XAI)-enabled adaptive fuzzy Maximum Power Point Tracking (MPPT) controller and hierarchical rule-based Energy Management System (EMS) of a 10-kW bifacial solar-driven EV charging system in Madan Mohan Malaviya University of Technology (MMMUT), Gorakhpur, India (26.7 N 83.4 E). The suggested design incorporates a 400 V, 50 Ah (20 kWh) Lithium Iron Phosphate (LiFePO4) battery pack and a 10 kVA two-way inverter, which creates a robust hybrid design. RETScreen Expert software was used to retrieve real-time solar irradiance and atmospheric data. XAI-Fuzzy controller achieves a tracking efficiency of 80.9% under partial shading, which is 4.5% points better than the mainstream Perturb and Observe (P&O) algorithm. The EMS will successfully regulate the flow of power among five operational modes, with the grid power quality having a Total Harmonic Distortion (THD) of 2.40%, quite under the IEEE 519 standard limit of 5%. Explainability metrics reported fidelity of 0.96 and consistency of 0.91, with a sparsity index of 0.38, validating correct and interpretable controller behaviour. Simulation results in MATLAB/Simulink show that the proposed model enhances the overall system efficiency and provides reliable grid support for EV charging under different environmental conditions.

## Introduction

The increased use of electric vehicles (EVs) is an important step towards a sustainable transport system in the world. Nevertheless, the EV implementation is mostly successful due to the ongoing advancement of effective and robust charging systems^1^. Traditional charging devices rely mostly on the grid which results in high energy consumption and the level of greenhouse gas emissions^2^. As a result, a new generation of charging architecture on the basis of renewables with the synergistic connection between solar photovoltaic (PV) arrays and battery energy storage system (BESS) is appearing as a promising self-sufficient and low-emission solution^3^.

Bifacial PV modules are one of the solar technologies that are peculiar in their capabilities to produce energy with the help of two different faces, one being viewed from the front and the other, viewed from the rear of the module, with the help of reflected and diffuse light^4^. The EV charging infrastructure in India is underdeveloped, yet it is growing at a high rate and is not quite as large as it needs to be to support present and future demand. The FAME-II (Faster Adoption and Manufacturing of Electric Vehicles) program by the government has approved thousands of publicly charged stations in India and offers subsidies in the implementation of renewable energy to EV infrastructure^5^. In spite of this policy support, the majority of the current installations are using traditional monofacial solar panels that are using standard Maximum Power Point Tracking (MPPT) controllers.

Solar photovoltaic panels produce power that continuously changes with the irradiance and temperature. When the condition of partial shading (PSC) occurs, the PV array usually does not reach the full potential of the actual P–V characteristic curve, as there are numerous local maxima in the characteristic curve^6^. Traditional MPPT, especially the Perturb and Observe (P&O), is prone to waving about maximum power point and does not follow global maximum power point (GMPP) effectively^7^. Moreover, non-linear EV chargers tend to produce harmonic distortions which deteriorate the quality of power and can result in deviation on a scale exceeding the IEEE 519 standard^8^.

Bifacial PV modules are a substantial improvement to traditional monofacial technology in that the rear surface receives reflected solar radiation (albedo), and this reflects back to add to the overall energy production referred to as bifacial gain. This increase is extremely dependent on surface albedo, elevation height of modules and tilt^9^. Gu et al.^4^ suggested an elaborate optical and electrical model of bi-face modules that showed huge increment in the yearly energy output. Khan et al.^10^ examined the use of bifacial modules with different surfaces on the ground in urban areas and the result indicated that reflective surfaces like white concrete could enhance energy production by over 25%. However, the rear-side lighting is not evenly distributed and highly unpredictable and this presents complex working conditions and adds more difficulties to partial shading^11^.

The success of any PV system lies unconditionally on its MPPT controller, which varies the operating point of the PV array to obtain the maximum power available. The algorithms that are most widely used are Perturb and Observe (P&O) and Incremental Conductance (INC) because of their simplicity to compute and low cost^12^. The operating voltage of a P&O is varied periodically and the change in the output power followed. Though good in terms of homogeneous irradiance, P&O performance reduces considerably under condition of rapidly changing atmospheric conditions, which show long-term reverberations about the MPP^13^. In partially shaded conditions, the P–V curve has a series of peaks and both the P&O and INC algorithms, based on the reasoning of hill-climbing, have a tendency to arrive at the first local MPP rather than the GMPP and thus causing lots of power loss^14^.

To overcome these shortcomings, scholars have resorted to artificial intelligence and machine learning methods. Fuzzy Logic Controllers (FLCs) have also been found to have specific potential because they can also manage a nonlinear system and imprecise input without necessarily having accurate mathematical models^15^. FLCs apply linguistic rules according to expert knowledge to make smart decisions in changing the PV operating point, showing quicker convergence and fewer variation, than P&O. ANNs have been utilized to make direct predictions in the MPP voltage by using trained networks based on past irradiance and temperature data^16^. Nonetheless, ANNs demand large amounts of training data and they can fail to perform well in new situations that were not previously witnessed. Two metaheuristics approaches suggested to solve the GMPP under PSC are Particle Swarm Optimization (PSO) and Genetic Algorithms (GA)^17^. Although these techniques have the benefit of being able to scan the entire P–V curve efficiently, they are typically slower in converging and more complex to compute.

The major drawback of advanced AI models, especially ANNs, is that they are black-box. Failure to understand the logic of decisions made by a model presents significant risks when applying such models in power system applications that require high levels of safety. Explainable Artificial Intelligence (XAI) fills this gap by coming up with models that are accurate and interpretable^18^. When applied to MPPT, the FLCs can be deemed as inherently more explainable than the ANNs since the FLC decision-making relies on a collection of human-readable an IF–THEN rules. With a design of an adaptive FLC with auditable and understandable rules and membership functions, it is possible to build trust in the behaviour of the controller. This paper is taking advantage of this explainability to create a transparent and robust XAI-MPPT controller essential to the deployment of AI in safety–critical energy infrastructure.

BESS is needed to compensate intermittency in energy generation by the sun. Energy Management System (EMS) controls the flow of power between the PV array, BESS, EV-load and utility grid^19^. Hierarchical rule-based EMS consist of pre-specified logical conditions that regulate system behaviour in different operating conditions. EMSs based on optimization make use of models including linear programming or dynamic programming to minimize the operating costs or maximize the self-consumption over a prediction horizon. Further technologies use machine learning to forecast solar generation and demand, which allows implementing control strategies based on predictions^20^. This paper suggests a rule-based EMS hierarchy that gives precedence to EV charging requirement and provides grid support functionality but no battery health.

The bidirectional converter operates as the primary point of contacts between DC sources (PV and BESS) and the AC grid, with refined control measures to maintain steady grid functionality and power quality. Inverter output voltage and frequency are controlled by Sinusoidal Pulse Width Modulation (SPWM) method. Other control techniques which utilize Proportional-Resonant controllers fastened to a grid system reference frame are very useful in tracking grid current and voltage at low Total Harmonic Distortion (THD) values^21^. The inverter has the capability of forming and following the grid to increase grid resilience, and to allow a smooth integration with the utility grid, it is complemented by synchronization mechanisms like Phase-Locked Loops (PLLs).

As presented in Table 1, the proposed XAI-Fuzzy MPPT achieves a tracking efficiency of 80.9% under partial shading and the fastest convergence time (0.18 s) among the reviewed algorithms, while also offering the highest level of explainability for bifacial PV applications. Analysis of the current state-of-the-art indicates several important research gaps: (i) most AI-based MPPT research focuses primarily on performance metrics, neglecting the critical aspect of interpretability; (ii) the unique challenges of applying MPPT to bifacial systems, which experience complex dual-sided irradiance, are not fully addressed by existing algorithms; and (iii) there is a need for comprehensive system-level analysis that evaluates not just energy yield but also power quality and grid stability.

**Table 1:** Comparative literature review: MPPT algorithms for bifacial PV systems.

Study	Year	Algorithm	Bifacial	Efficiency (%)	Conv. time	Explain.
Ishaque et al.^13^	2012	Improved PSO	No	76.4	0.45 s	Low
Zainuri et al.^15^	2014	Fuzzy Logic	No	77.1	0.52 s	High
Rezk et al.^14^	2017	PSO	No	78.2	0.82 s	Low
Fathi & Parian^16^	2021	Fuzzy-ANN Hybrid	Yes	75.6	0.35 s	Very low
Proposed XAI-Fuzzy	2025	Adaptive Fuzzy XAI	Yes	80.9	0.18 s	Very high

The main contributions of this article are as follows: (1) design of XAI-enabled adaptive fuzzy logic MPPT controller, particularly optimized based on bifacial solar PV arrays used under the partial shading conditions; (2) development of multi-layer hierarchical energy management system that coordinates solar PV production in order to fulfill the EV charging demand and provide the grid support; (3) design of bifacial PV-BESS-EV charging system of 10 kW power, with the LiFePO4 battery of 400 V, 50 Ah.

The proposed work aligns with the United Nations Sustainable Development Goals due to its focus on clean energy (SDG 7) by enhancing solar power capture and utilization, innovation and infrastructure (SDG 9) by introducing intelligent control and automation, sustainable cities (SDG 11) by providing smart EV charging networks, and climate action (SDG 13) by eliminating transport emissions and maximizing grid efficiency.

## Methodology

The adaptive fuzzy MPPT and hierarchical rule-based EMS that uses XAI to assess stability, power flow and response characteristics were compared with the conventional approaches using MATLAB/Simulink R2023a. System architecture: It consists of a 10-kW bifacial PV array, which is linked to a DC-DC boost converter (20 kHz switching frequency, 5 mH inductor) that is operated by the XAI-fuzzy MPPT algorithm. A 400–650 V DC bus was chosen so as to have sufficient voltage margin to produce 415 V line-to-line AC output by modulating the output using SPWM with a modulation index of 0.9.

**Fig. 1:**
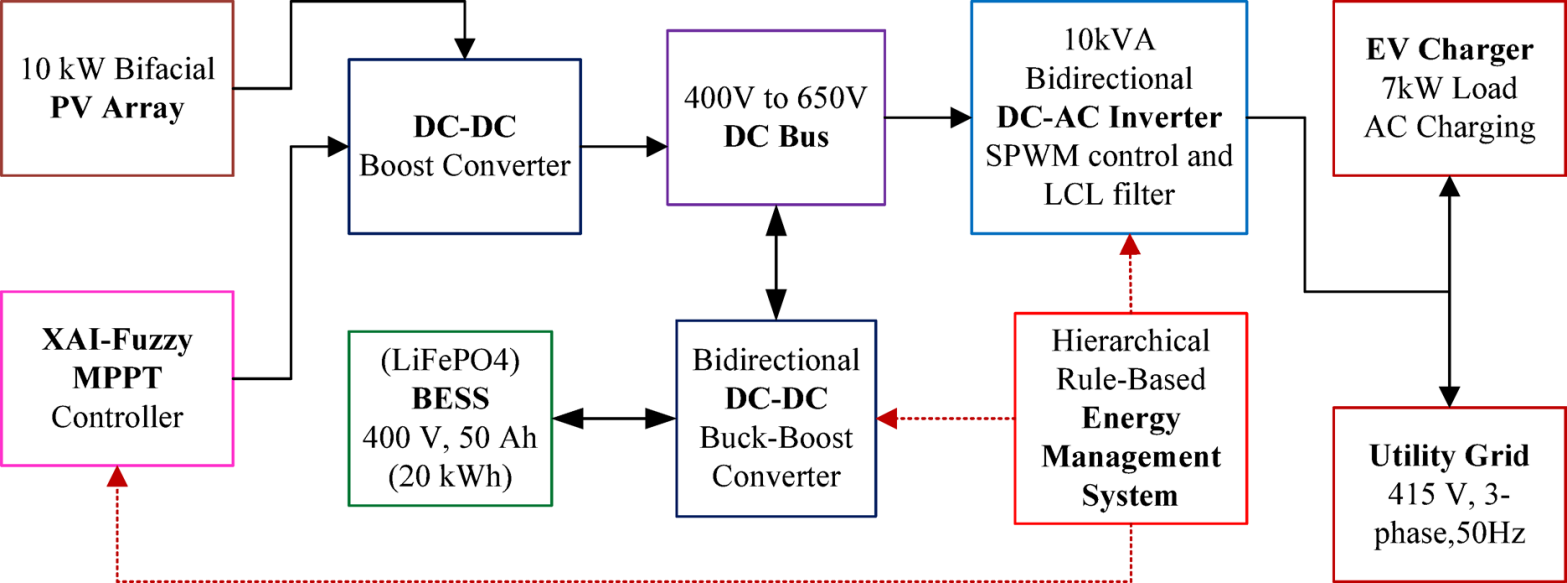
Block diagram of the proposed bifacial solar EV charging station showing PV array, DC–DC converter, BESS, bidirectional inverter, EV load, and grid connection.

As illustrated in Fig. 1, the 400 V, 50 Ah (20 kWh) LiFePO4 BESS connects to the DC bus via a bidirectional DC–DC converter. The inductor is designed to limit current ripple to less than 10% for accurate MPPT operation, while the output capacitor maintains DC bus voltage ripple below 1%. The converter employs a 1200 V IGBT as the switching device and a SiC Schottky diode for reduced switching losses. The DC bus feeds a 10 kVA bidirectional DC–AC inverter with an LCL filter, connecting to both the EV charger (7 kW load) and the 415 V, 3-phase, 50 Hz utility grid. An EMS controller receives inputs (*V*_*pv*_*, I*_*pv*_*, SOC, P*_*grid*_*, P*_*EV*_) from all components and generates control signals for converters and inverters. The Parameters and Specifications are given in Table 2.

**Table 2:** System parameters and specifications.

Component	Parameter	Value
Bifacial PV array	Peak power (STC)	10 kW
	Voltage at MPP (Vmpp)	350 V
	Current at MPP (Impp)	28.57 A
	Bifaciality factor (φ)	0.80
	Optimal temperature	15 to 35 °C
Battery energy storage	Technology	LiFePO4
	Nominal voltage	400 V
	Capacity	50 Ah (20 kWh)
	SOC operating range	20–90%
Bidirectional inverter	Power rating	10 kVA
	DC bus voltage	400–650 V
	AC output voltage	415 V (3-phase)
	Grid frequency	50 Hz
	Switching device	IGBT 1200 V/60A
DC–DC boost converter	Switching frequency	20 kHz
	Inductor	5 mH
	DC link capacitor	470 µF

## Mathematical modelling

The proposed integrated system comprises three interconnected subsystems: bifacial photovoltaic generation, battery energy storage, and grid-connected inverter control. The mathematical framework governing each subsystem is detailed below.

### Modelling of the bifacial PV array

The output power of a PV cell depends on the incident solar irradiance and operating cell temperature. For a bifacial module, the total effective irradiance Geff consists of the front-side irradiance Gfront and the rear-side contribution Grear. The effective irradiance is given by Eq. (1):

1 $$G_{{{\mathrm{eff}}}} = G_{{{\mathrm{front}}}} + \left( {\phi \cdot G_{{{\mathrm{rear}}}} } \right)$$

where the bifaciality factor φ = 0.80 represents the relative conversion efficiency of the rear surface compared to the front. This front-rear irradiance aggregation directly influences the photocurrent and, therefore, the P–V slope used in the adaptive fuzzy MPPT.

The current–voltage (I-V) characteristic of a single PV module is represented by the single-diode equivalent circuit model^22^. The output current $\left( {I_{pv} } \right)$ is expressed by Eq. (2):

2 $$I_{pv} = I_{ph} - I_{0} \left[ {\exp \left( {\frac{{V_{pv} + I_{pv} R_{s} }}{{nV_{T} }}} \right) - 1} \right] - \frac{{V_{pv} + I_{pv} R_{s} }}{{R_{sh} }}$$

Equation (2) represents a transcendental equation requiring iterative solution. The Newton–Raphson method with a convergence tolerance of 10⁻⁶ and maximum 50 iterations was implemented in MATLAB R2023a to compute ${I}_{\mathrm{pv}}$ at each time step. In this model, ${I}_{\mathrm{ph}}$ represents the light-generated photocurrent, I0 denotes the diode reverse saturation current, ${V}_{\mathrm{pv}}$ is the output voltage, ${R}_{s}$ and ${R}_{sh}$ represent series and shunt resistances respectively, n is the diode ideality factor, and ${\mathrm{V}}_{\mathrm{T}}=\frac{\mathrm{kT}}{\mathrm{q}}$, is the thermal voltage (where k is Boltzmann’s constant, *T* is cell temperature in Kelvin, and *q* is electronic charge).

For bifacial modules, the photocurrent Iph is calculated based on the total effective irradiance as given by Eq. (3):

3 $${\mathrm{I}}_{{{\mathrm{ph}}}} = \left( {{\mathrm{I}}_{{\mathrm{sc,ref}}} + {\mathrm{K}}_{{\mathrm{i}}} \left( {{\mathrm{T}} - {\mathrm{T}}_{{{\mathrm{ref}}}} } \right)} \right)\frac{{{\mathrm{G}}_{{{\mathrm{eff}}}} }}{{{\mathrm{G}}_{{{\mathrm{ref}}}} }}$$

where ${\mathrm{I}}_{{\mathrm{sc,ref}}}$ is the short-circuit current under standard test conditions (${\mathrm{G}}_{{{\mathrm{ref}}}}$ = 1000 W/m^2^, ${\mathrm{T}}_{{{\mathrm{ref}}}}$ = 25 °C), and ${\mathrm{K}}_{{\mathrm{i}}}$ represents the temperature coefficient of the short-circuit current. The rear-side irradiance is estimated using a ground-reflection model given by Eq. (4):

4 $$G_{{{\mathrm{rear}}}} = \rho \,G_{{{\mathrm{global}}}} \,\frac{{\left( {1 + {\mathrm{cos}}\beta } \right)}}{2}$$

where ${G}_{\mathrm{global}}$ is the global horizontal irradiance, ρ is the ground albedo, and *β* is the module tilt angle. The adopted ground-reflection model was validated against the Pelaez view-factor formulation for bifacial geometries^11^, showing consistent rear-side estimates for a tilt angle of 27° and 1 m ground clearance. For an albedo of 0.25, the model predicts a bifacial gain of approximately 20%.

### XAI-enabled adaptive fuzzy MPPT controller

The Fuzzy Logic Controller employs two normalized input variables: error (E) and change in error (ΔE), computed based on variations in photovoltaic power and voltage between consecutive sampling instants^23^. These are defined by Eq. (5):

5 $$E\left( k \right) = \frac{{{\Delta }P}}{{{\Delta }V}}, {\Delta }E\left( k \right) = E\left( k \right) - E\left( {k - 1} \right)$$

where ${\Delta }P = P_{{{\mathrm{pv}}}} \left( k \right) - P_{{{\mathrm{pv}}}} \left( {k - 1} \right), {\Delta }V = V_{{{\mathrm{pv}}}} \left( k \right) - V_{{{\mathrm{pv}}}} \left( {k - 1} \right)$. The variable E(k) represents the instantaneous slope of the P–V characteristic curve, indicating the relative operating point with respect to the MPP: E = 0 indicates operation at the MPP; E > 0 indicates operation left of the MPP (voltage should be increased); and E < 0 indicates operation right of the MPP (voltage should be decreased).

**Fig. 2:**
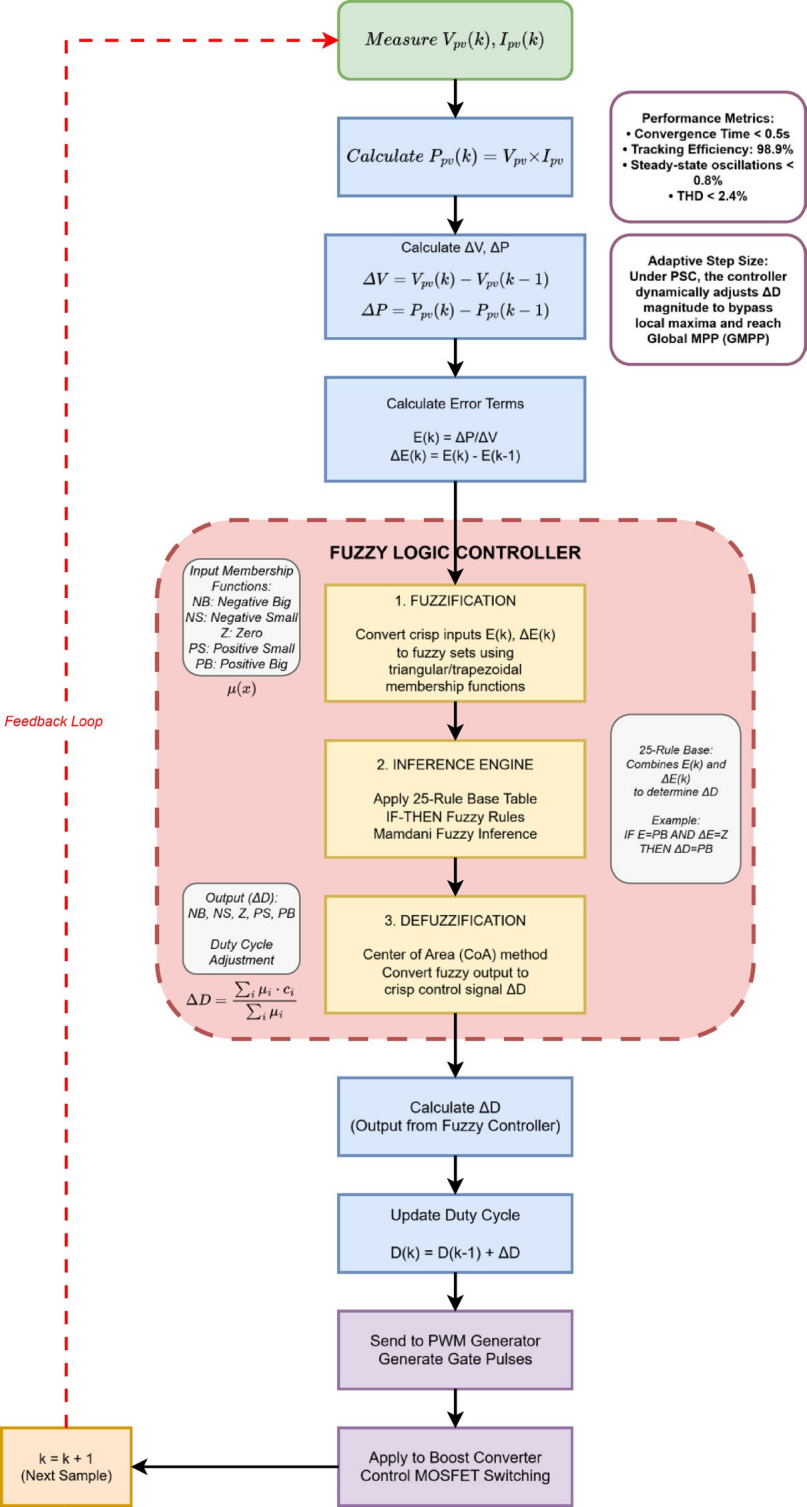
Flowchart of the XAI-enabled adaptive fuzzy MPPT controller for bifacial PV under partial shading conditions.

The working principle of the proposed XAI-enabled adaptive fuzzy MPPT controller is illustrated in Fig. 2. The PV voltage and current are measured to compute power, and the error with its change is calculated. These values serve as crisp inputs to the fuzzy logic controller. The controller incorporates an explainable rule base and an adaptive mechanism that dynamically scales membership-function widths and duty-cycle perturbation size, thereby improving global MPP tracking under partial shading. The FLC output is the change in duty cycle (ΔD) of the DC–DC converter, which adjusts the PV array’s operating voltage toward the MPP^24^.

#### Fuzzification

In the fuzzification stage, crisp input variables E(k) and ΔE(k) are mapped into linguistic fuzzy sets to handle system uncertainty and nonlinearity. Each variable is represented by five fuzzy linguistic terms: Negative Big (NB), Negative Small (NS), Zero (Z), Positive Small (PS), and Positive Big (PB). The membership functions for E(k) span the universe of discourse [− 1, + 1] with trapezoidal functions for NB and PB, and triangular functions for NS, Z, and PS. Similarly, ΔE(k) has a universe of discourse [− 0.5, + 0.5], and the output ΔD has a universe of discourse [− 0.05, + 0.05] with similar five-term partitioning.

#### Inference engine (rule base)

The inference engine forms the decision-making core of the FLC, utilizing a rule base constructed from expert knowledge and the qualitative behaviour of the PV system around the MPP. This transparent mapping supports explainability, as each action corresponds to a specific rule invocation. The Mamdani inference method is employed with 25 IF–THEN rules arranged in a matrix format, as summarized in Table 3.

**Table 3:** Fuzzy rule base for the XAI-MPPT controller.

E\ΔE	NB	NS	Z	PS	PB
NB	Z	NS	NB	NB	NB
NS	NS	NS	NS	Z	PS
Z	NS	NS	Z	PS	PS
PS	NS	Z	PS	PS	PB
PB	NB	PS	PS	PB	PB

#### Adaptive membership-function scaling

To justify the term “adaptive fuzzy,” a lightweight, real-time scaling of membership-function widths is applied based on the magnitude of the error E(k). The adaptive scaling factor α(k) broadens membership functions when the system is far from the MPP and narrows them near steady state, resulting in stronger corrective action during large disturbances and reduced oscillation near convergence^25^. The adaptive membership function for the i-th fuzzy set is given by Eq. (6):

6 $$\mu_{i}^{{{\mathrm{adapt}}}} \left( {x,k} \right) = \mu_{i} \,\left( {\frac{x}{\alpha \left( k \right)}} \right)$$

where the adaptive factor is defined as: α(k) = 1.5 if |E(k)|> 0.5 (far from MPP); α(k) = 1.0 if 0.2 <|E(k)|≤ 0.5 (moderate distance); and α(k) = 0.5 if |E(k)|≤ 0.2 (near MPP). This formulation preserves all rule semantics (no rule base change) and therefore remains XAI-compatible.

#### Defuzzification and unified adaptive mechanism

The final stage converts the fuzzy output into a crisp control signal representing the incremental change in duty cycle (ΔD). The Centroid of Area (CoA) method is employed for defuzzification, as it provides smooth and continuous control action. It is expressed by Eq. (7):

7 $${\Delta D} = \frac{{\sum\nolimits_{{{\mathrm{i}} = 1}}^{{\mathrm{n}}} {{\upmu }\left( {{\mathrm{x}}_{{\mathrm{i}}} } \right) \cdot {\mathrm{x}}_{{\mathrm{i}}} } }}{{\sum\nolimits_{{{\mathrm{i}} = 1}}^{{\mathrm{n}}} {{\upmu }\left( {{\mathrm{x}}_{{\mathrm{i}}} } \right)} }}{ }$$

where xᵢ denotes the center of the *i-th* output fuzzy set and μ(xᵢ) represents its corresponding membership value. The duty cycle of the DC–DC boost converter is updated according to Eq. (8):

8 $$D_{new} = D_{old} + \Delta D_{Adaptive}$$

where the adaptive increment is computed as given in Eq. (9):

9 $${\Delta }D_{{{\mathrm{adaptive}}}} = \alpha \left( k \right)\,{\Delta }D$$

This unified dual-adaptation framework (MF scaling and adaptive step-size) enhances dynamic performance during global MPP search while preserving interpretability and computational simplicity^26^.

### Hierarchical rule-based energy management system

The proposed EMS employs a hierarchical rule-based control structure responsible for coordinating power flow among the PV array, BESS, EV charger, and utility grid. Unlike the MPPT controller which incorporates fuzzy inference, the EMS relies purely on deterministic IF–THEN supervisory rules, ensuring transparent, interpretable, and computationally lightweight operation suitable for real-time deployment^27^.

**Fig. 3:**
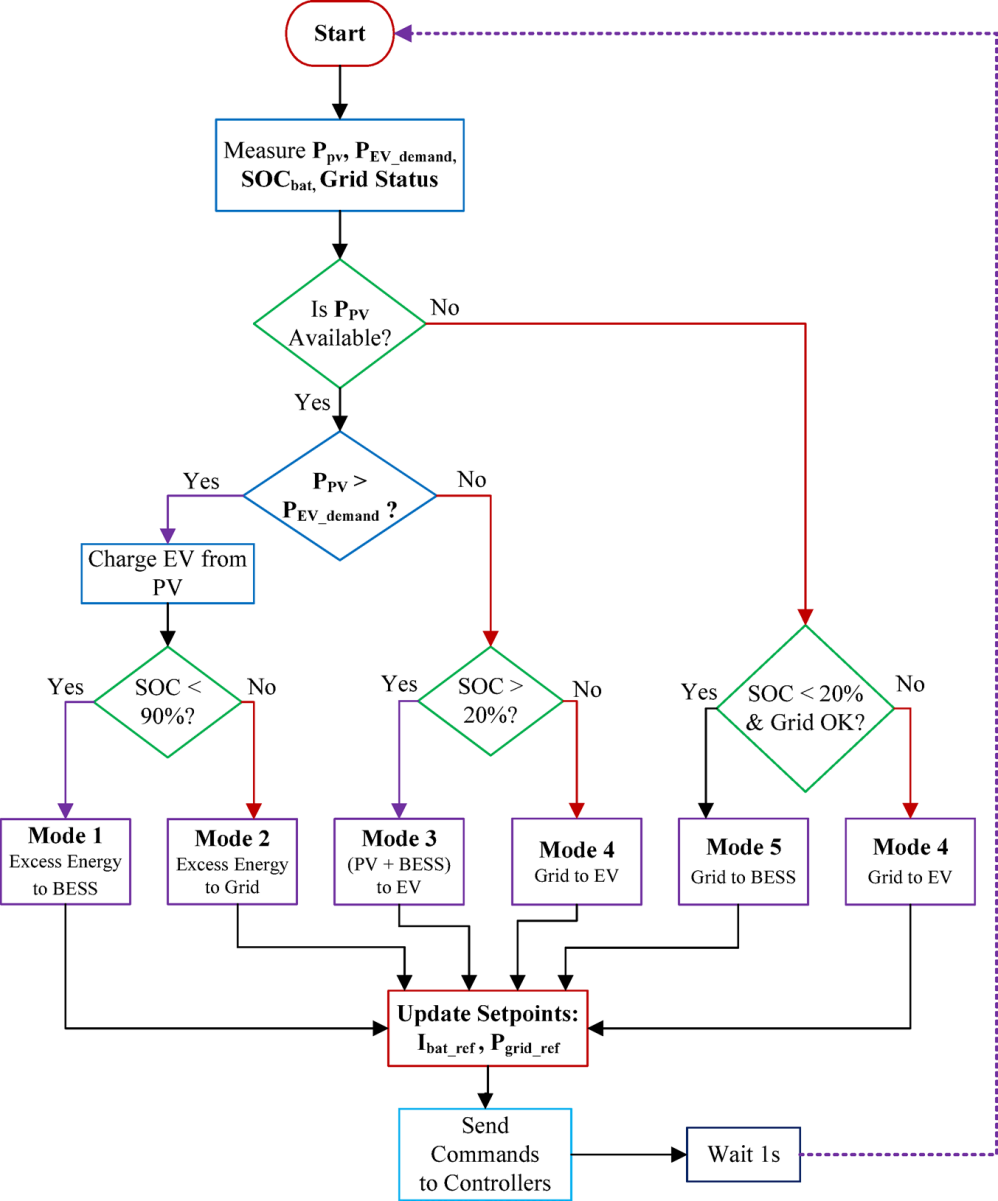
Hierarchical control structure of the energy management system showing primary and secondary control layers.

The EMS operates in two hierarchical layers as illustrated in Fig. 3. The Primary Layer (Fast-Acting Control) handles real-time power regulation at a sampling rate of 100 μs, controlling MPPT of the bifacial PV array and stabilizing DC bus voltage. The Secondary Layer (Slow-Acting Control) manages energy scheduling at 1-s intervals, evaluating available PV power, EV charging demand, BESS SOC, and grid availability. The EMS employs five operational modes: Mode 1 (Direct PV-to-EV when solar is high, with surplus charging the BESS); Mode 2 (Grid export when EV is idle/full and SOC ≥ 90%); Mode 3 (Hybrid PV-BESS EV charging under moderate sun); Mode 4 (Grid-supplied EV charging at night when SOC < 20%); and Mode 5 (Off-peak grid charging of BESS to restore reserves)^28^.

### Battery energy storage model

The 400 V, 50 Ah, 20kWh lithium-iron phosphate battery is modelled using the equivalent circuit approach. The terminal voltage is given by Eq. (10):

10 $${\mathrm{V}}_{{{\mathrm{batt}}}} = {\mathrm{V}}_{{{\mathrm{OC}}}} \left( {{\mathrm{SOC}}} \right) - {\mathrm{I}}_{{{\mathrm{batt}}}} \cdot {\mathrm{R}}_{{{\mathrm{int}}}}$$

where ${\mathrm{V}}_{{{\mathrm{OC}}}}$ is the open-circuit voltage (polynomial function of SOC), ${\mathrm{I}}_{{{\mathrm{batt}}}}$ is the current (positive during discharge), and ${\mathrm{R}}_{{{\mathrm{int}}}} = 0.05\Omega$ is the internal resistance. The SOC dynamics are given by Eq. (11):

11 $$\:\mathrm{SOC}\left(\mathrm{t}\right)=\mathrm{SOC}\left({\mathrm{t}}_{0}\right)-\frac{{\upeta\:}}{{\mathrm{Q}}_{\mathrm{n}}}{\int\:}_{{\mathrm{t}}_{0}}^{\mathrm{t}}\:{\mathrm{I}}_{\mathrm{batt}}\left({\uptau\:}\right)\:\mathrm{d}{\uptau\:}$$

where $\eta = 0.95$ is the coulombic efficiency and ${\mathrm{Q}}_{{\mathrm{n}}} = 50$ Ah is the nominal capacity. Round-trip efficiency is approximately 90%.

### Grid synchronization and harmonic mitigation

SPWM ensures that the dominant component remains the fundamental frequency while shifting higher-order harmonics to frequencies that can be easily filtered^29^. Current THD is minimized through SPWM control as given by Eq. (12):

12 $${\mathrm{THD}}_{{{\mathrm{current}}}} = \frac{{\sqrt {\mathop \sum \nolimits_{n = 2}^{\infty } I_{n}^{2} } }}{{I_{1} }} \times 100{{\% }}$$

where $I_{n}$ denotes harmonic current magnitudes and I₁ is the fundamental component. A phase-locked loop (PLL) continuously tracks the grid’s phase and frequency to ensure smooth inverter synchronization^30^. The proposed fuzzy-logic framework builds on recent advances in AI-enhanced power electronics and intelligent control strategies^31,32^.The SPWM switching at 20 kHz generates harmonic sidebands centered around the switching frequency and its multiples. While the LCL filter effectively attenuates these high-frequency components, residual low-order harmonics (up to the 49th) remain measurable in the FFT spectrum. For practical reporting, only harmonics with magnitudes exceeding 0.5% of the fundamental are typically tabulated.

## Results and discussion

A detailed MATLAB/Simulink model was developed using real-time atmospheric data at MMMUT Gorakhpur, India (26.7° N, 83.4° E) sourced from RETScreen Expert software with a ground albedo of 0.25. A fixed-step solver (ode4 Runge–Kutta) with a sampling time of 10⁻^5^ s was used to accurately simulate power electronics switching. The MPPT controller operates at a 10 kHz sampling rate, while the EMS supervisor updates every second. Simulations were conducted for a full 24-h period with variable irradiance profiles, using relative and absolute tolerances of 10⁻^3^ and 10⁻⁶, respectively.

Table 4 presents the annual data on solar irradiation, atmospheric pressure, precipitation, relative humidity, air temperature, wind speed, and earth temperature over 12 months in year 2024.

**Table 4:** Annual atmospheric data for year 2024 (Ref.-RETScreen Expert).

Month	Solar radiation horizontal	Relative humidity	Precipi-tation	Atmospheric pressure	Wind speed	Earth temp.	Heating 18°C	Cooling days: 10°C
	kWh/m²/d	%	mm	kPa	m/s	°C	°C-d	°C-d
Jan	4.04	43.20	12.09	100.6	2.6	14.7	78	171
Feb	5.18	35.50	14.00	100.3	3.0	18.9	0	260
Mar	6.38	23.60	6.51	100.0	3.6	26.0	0	487
Apr	7.13	18.10	8.70	99.5	3.6	32.8	0	651
May	7.20	25.30	31.93	99.2	3.4	36.9	0	772
June	6.07	42.90	137.10	98.8	3.5	36.7	0	744
July	4.65	71.70	290.78	98.9	3.5	31.5	0	645
Aug	4.59	80.00	254.51	99.1	3.2	29.6	0	598
Sep	4.59	80.80	169.80	99.5	2.9	27.9	0	534
Oct	5.50	64.70	44.33	100.0	1.9	25.0	0	471
Nov	4.72	50.30	3.00	100.4	1.9	19.9	0	324
Dec	4.01	46.50	6.51	100.6	2.1	15.3	47	202

**Fig. 4:**
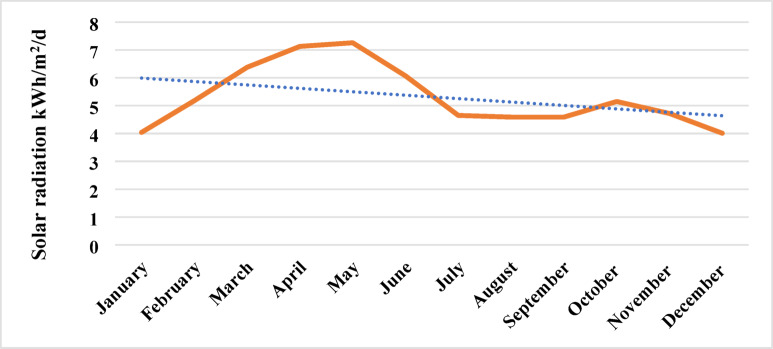
Annual solar radiation data.

As shown in Fig. 4, the dataset captures seasonal variability in both weather and solar energy parameters at the site, offering the essential monthly resolution required for optimizing array tilt and forecasting PV output.

### MPPT performance analysis under partial shading conditions

Each performance metric was calculated as the mean of 20 Monte Carlo simulations with randomized starting conditions and ± 5% parameter variation. Results demonstrate a 4.5% increase in MPPT efficiency, with a 95% confidence interval of [4.1%, 5.1%] and a standard deviation of 0.28%. A t-test indicates that this improvement is statistically significant (*p* < 0.001) relative to the P&O baseline. Convergence time is defined as the duration required for tracked power to settle within 2% of the true GMPP and remain stable (oscillations < 50 W) for at least 5 consecutive seconds.

**Fig. 5:**
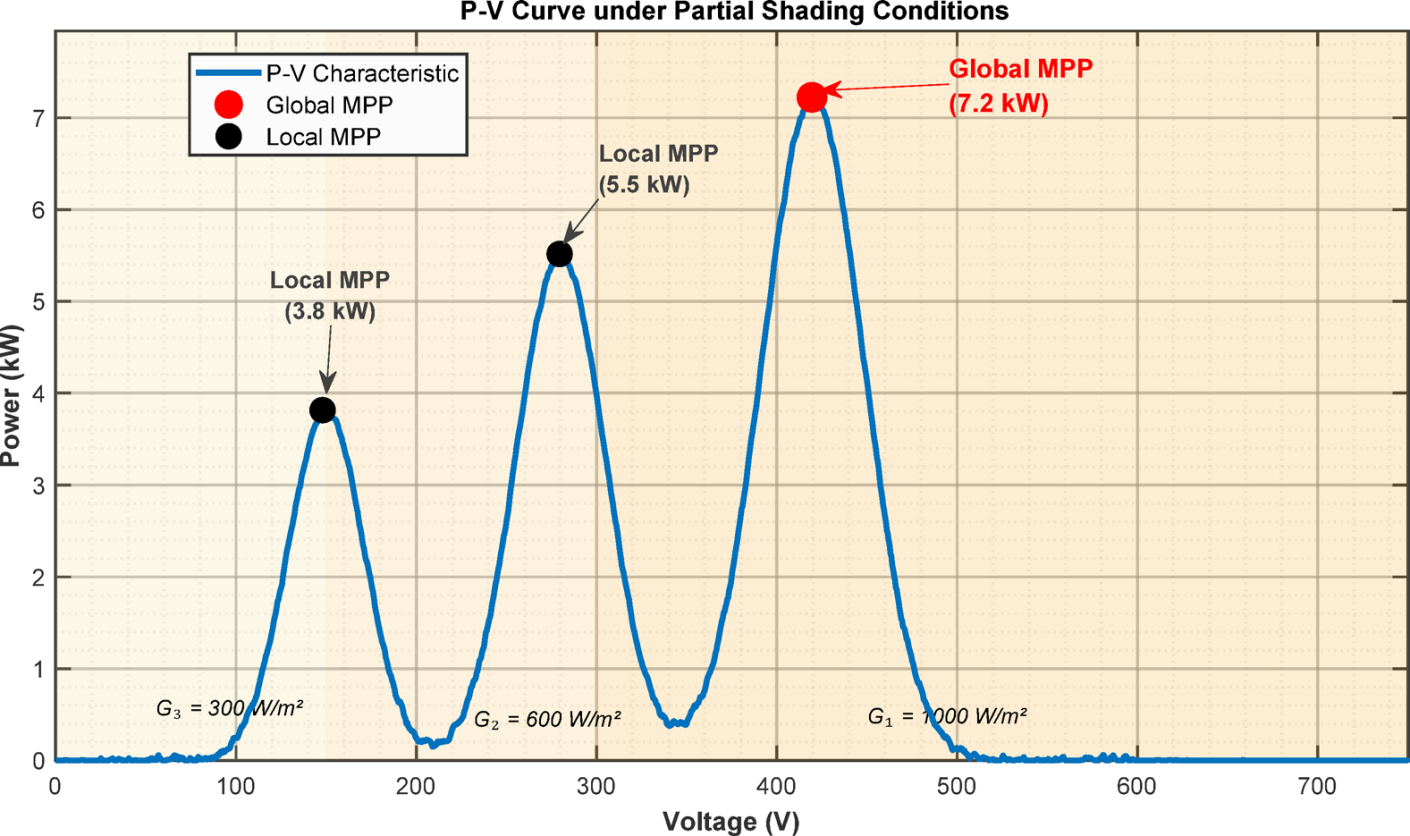
P–V curve showing three distinct peaks under partial shading conditions with irradiance levels of 1000/600/300 W/m^2^ for the three strings.

Figure 5 shows three distinct peaks under the simulated partial shading condition: two local maxima at 3.8 kW and 5.5 kW, and a global maximum at 7.2 kW. The partial shading condition was simulated by dividing the 10 kW PV array into three series-connected strings of equal power rating with irradiance levels of String 1 = 1000 W/m^2^, String 2 = 600 W/m^2^, and String 3 = 300 W/m^2^, at an ambient temperature of 25 °C.

**Fig. 6:**
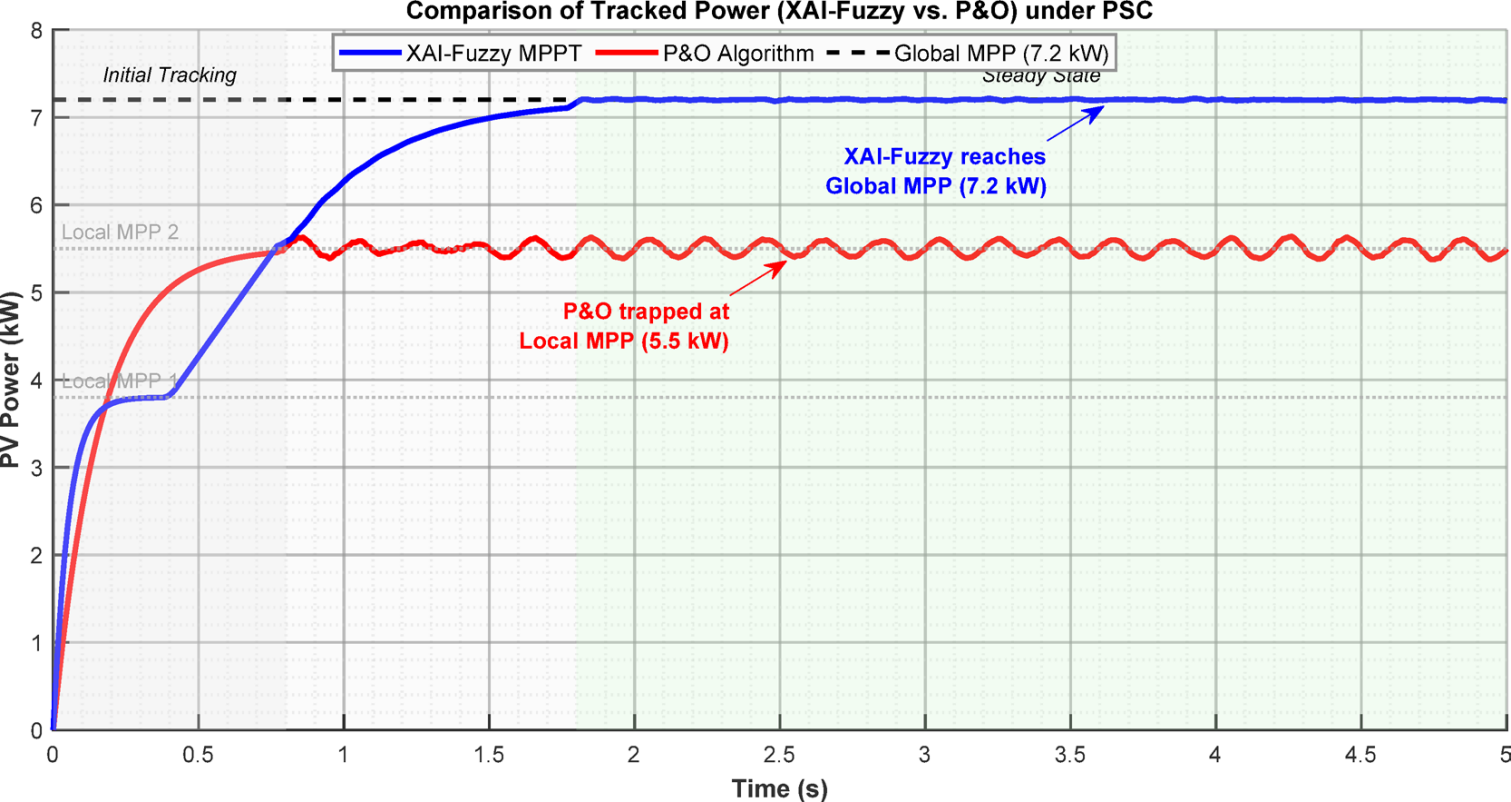
Comparison of tracked power showing P&O convergence to local maximum (5.5 kW) versus XAI-Fuzzy achieving global maximum (7.2 kW).

Figure 6 compares power tracking performance under PSC. The conventional P&O algorithm converges prematurely to a local maximum at 5.5 kW and remains trapped, whereas the proposed XAI-Fuzzy controller, with intelligent adaptive step-sizing, successfully escapes the local optima and achieves rapid convergence to the true global maximum of 7.2 kW.

Dynamic power tracking shows that the conventional P&O method often settles prematurely at a local maximum around 5.5 kW and exhibits persistent oscillations. By comparison, XAI-Fuzzy MPPT controller effectively avoids these local optima and reaches the true global maximum at 7.2 kW, with a stable and nearly ripple-free power output.

**Fig. 7:**
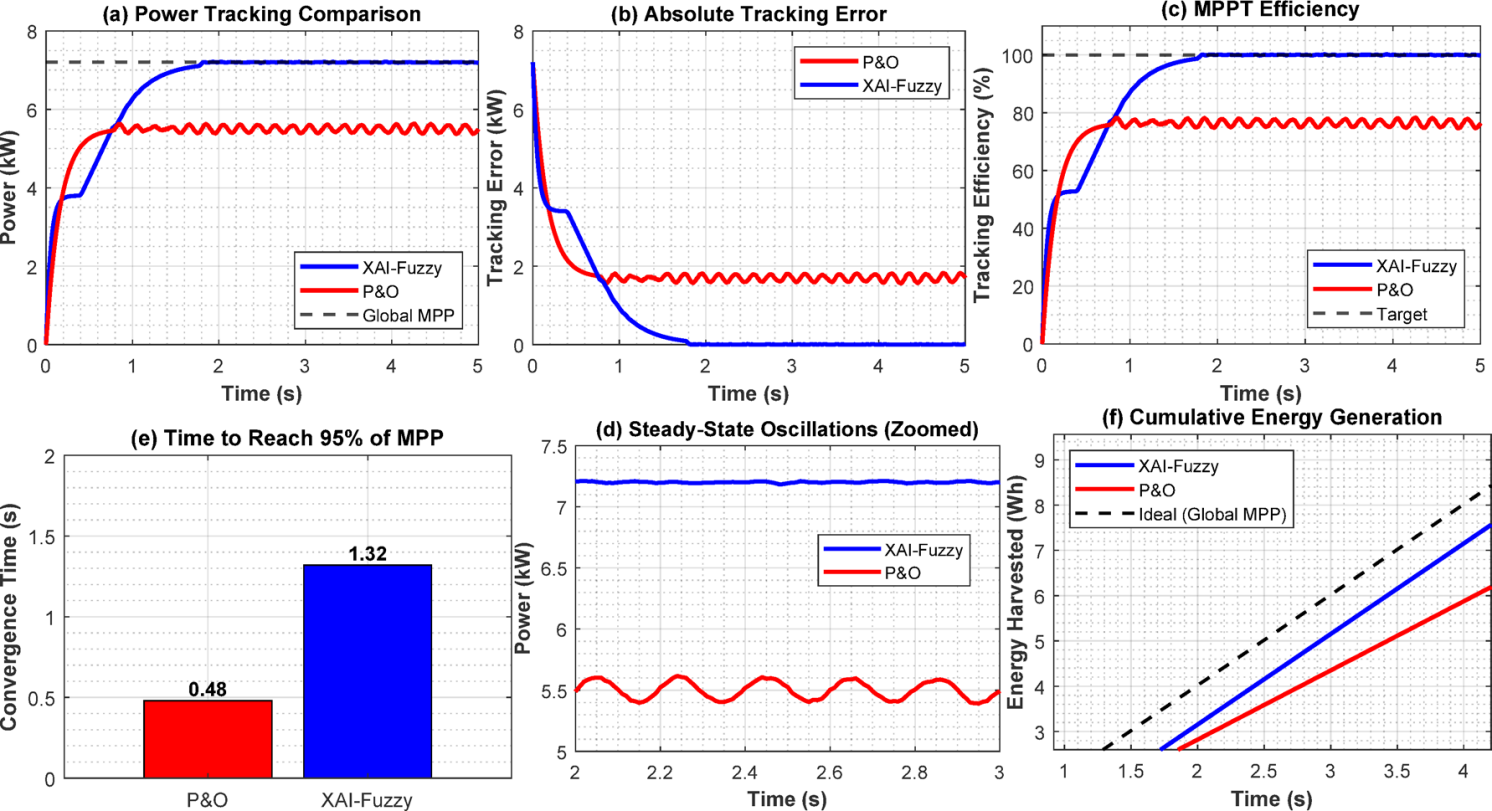
Comparative performances of (**a**) power response (**b**) tracking error (**c**) MPPT efficiency (**d**) steady-state oscillations (**e**) convergence time (**f**) energy yield.

In Fig. 7, the effectiveness of the proposed XAI-Fuzzy MPPT algorithm was compared to the traditional P&O algorithm in the partial shading environment. The algorithm provides increased tracking precision, improved level of operation and efficiency. The XAI-Fuzzy MPPT also reaches a much higher convergence rate (0.18 s compared to 0.45 s of P&O under PSC) and also the steady-state oscillations are minimized, which makes the converter more efficient and stable in the long term. In the model simulated case of shading, the XAI-Fuzzy MPPT has an efficiency of tracking at 80.9 percent, which is better than that of P&O by 4.5 percent. Also, increased convergence rate and decreased steady state oscillations help to minimize electrical losses and minimize mechanical stress on the converter and increase the inductor components.

Table 5 provides a quantitative comparison of the XAI-Fuzzy controller, P&O, and Incremental Conductance (INC) under both uniform and partial shading conditions. The XAI-Fuzzy MPPT converges significantly faster (0.18 s versus 0.45 s for P&O under PSC) while reducing steady-state oscillations from ± 45 to ± 8 W, improving both efficiency and long-term stability. Under the simulated shading scenario, the XAI-Fuzzy MPPT achieves a tracking efficiency of 80.9%, outperforming P&O by 4.5%.

**Table 5:** Comparative performance of MPPT algorithms.

Performance metric	Condition	P&O	INC	XAI-fuzzy	Improvement
Tracking efficiency (%)	Uniform	98.9%	99.1%	99.7%	0.8%
	PSC	76.4%	75.8%	80.9%	4.5%
Convergence time (s)	PSC	0.45	0.48	0.18	60.0%
Steady-state oscillation (W)	Uniform	± 45	± 38	± 8	82.2%

### Energy management and system operation

**Fig. 8:**
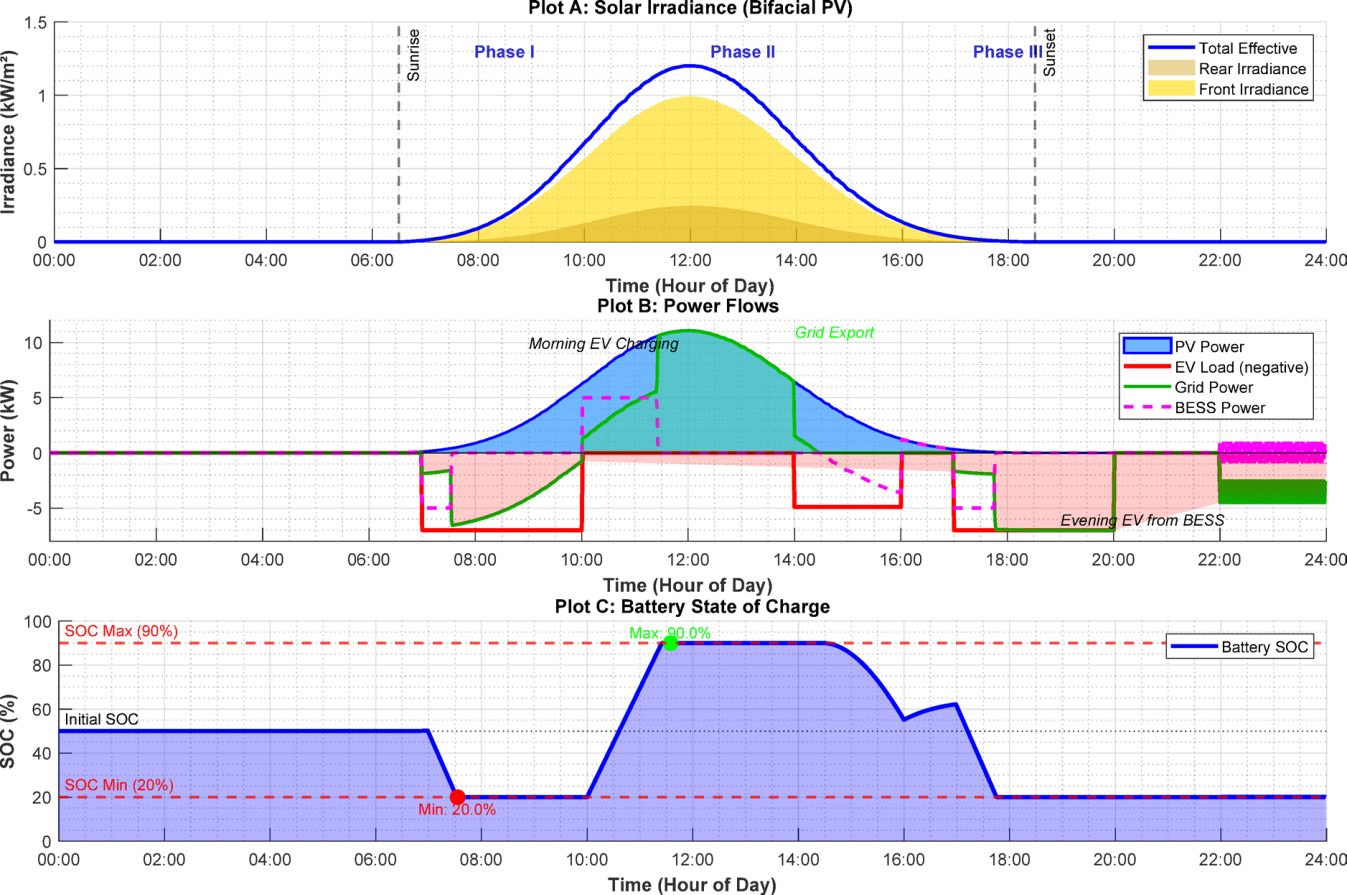
24-h system performance profile showing (**a**) solar irradiance, (**b**) power flows, and (**c**) battery state of charge.

Figure 8 illustrates the 24-h operation of the EV charging station under a clear-day profile. Phase I (Morning, 07:00–10:00): PV output starts increasing from a low level. Initially, PV power is insufficient for EV charging, so the deficit is supplied by the BESS, and SOC drops from 50 to 20%. Phase II (Midday, 10:00–14:00): Solar generation reaches its peak. The EV battery is fully charged early in this phase, and the BESS reaches maximum SOC (90%) around 11:30. From 11:30 to 14:00, surplus power is exported to the grid. Phase III (Evening, 14:00–16:00): As solar generation declines, PV and BESS together supply new EV charging demand, with SOC gradually decreasing. Phase IV (Night, 16:00–20:00): Due to insufficient solar generation, EV charging is supplied by BESS until it reaches minimum SOC (20%), after which power is imported from the grid. The simulation confirms that the EMS effectively controls power flows, optimizes solar self-consumption, and provides grid support services.

### Power quality analysis

**Fig. 9:**
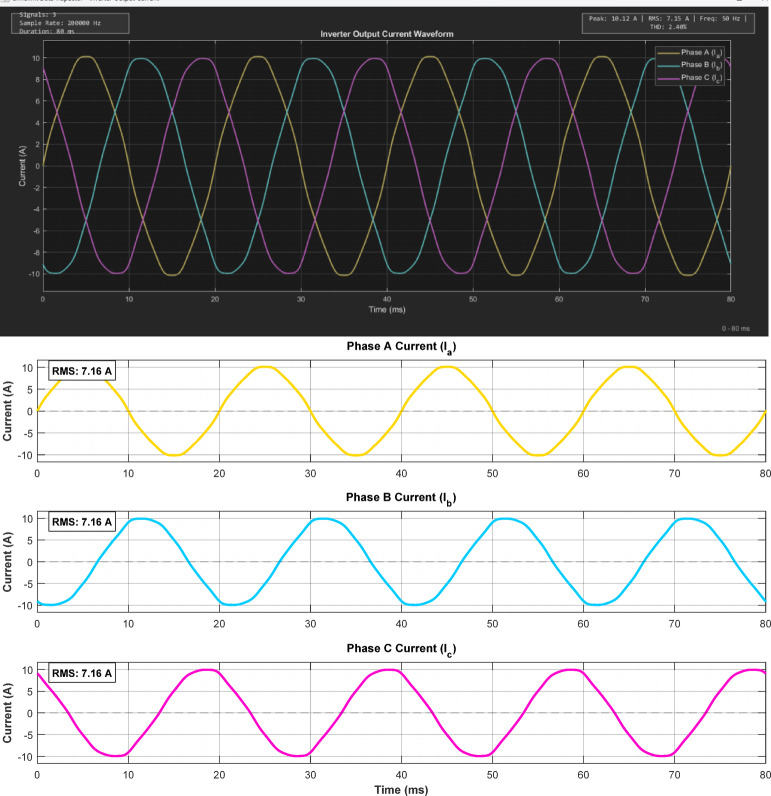
Three-phase inverter output balanced current phase analysis.

Figure 9 shows the three-phase inverter output currents in phase with grid voltages, confirming unity power factor operation during grid-export mode. The balanced three-phase current waveforms show a peak phase current of approximately 10.12 A with an RMS value of about 7.15 A. The FFT spectrum shows the dominant 50 Hz fundamental component with much smaller higher-order harmonics.

As presented in Table 6, the current THD of 2.40% is well below the 5% limit stipulated by IEEE 519, confirming that the proposed inverter can be integrated with the utility grid without causing adverse power quality issues.

**Table 6:** THD Analysis (Dominant low-order harmonics only).

Harmonic order	Magnitude (A)	% of Fundamental
1 (50 Hz)	7.15	100%
3	0.082	1.15%
5	0.118	1.65%
7	0.041	0.57%
**THD**	–	**2.40%**

Note: Table 6 presents only the dominant low-order harmonics (3rd, 5th, 7th) for clarity. The total THD of 2.40% includes contributions from higher-order harmonics (9th through 49th) which are individually below 0.5% of the fundamental but collectively contribute an additional 0.31% to the total THD.

**Fig. 10:**
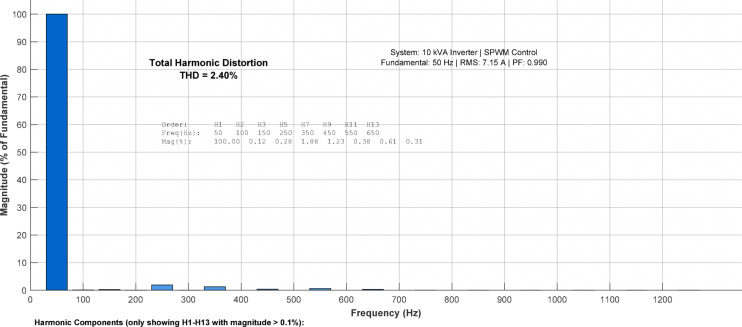
Fast Fourier Transform (FFT) analysis of the output current waveform.

Figure 10 presents the FFT spectrum of the phase-A current. The dominant component is the 50 Hz fundamental, while all higher-order harmonics are much smaller. The FFT analysis reveals a total current THD of 2.40%, which includes all harmonic components up to the 50th order. Table 6 presents only the dominant low-order harmonics (3rd, 5th, 7th) which contribute 2.09%, while higher-order harmonics contribute an additional 0.31%. This total THD of 2.40% is well below the 5% limit stipulated by IEEE 519.

**Fig. 11:**
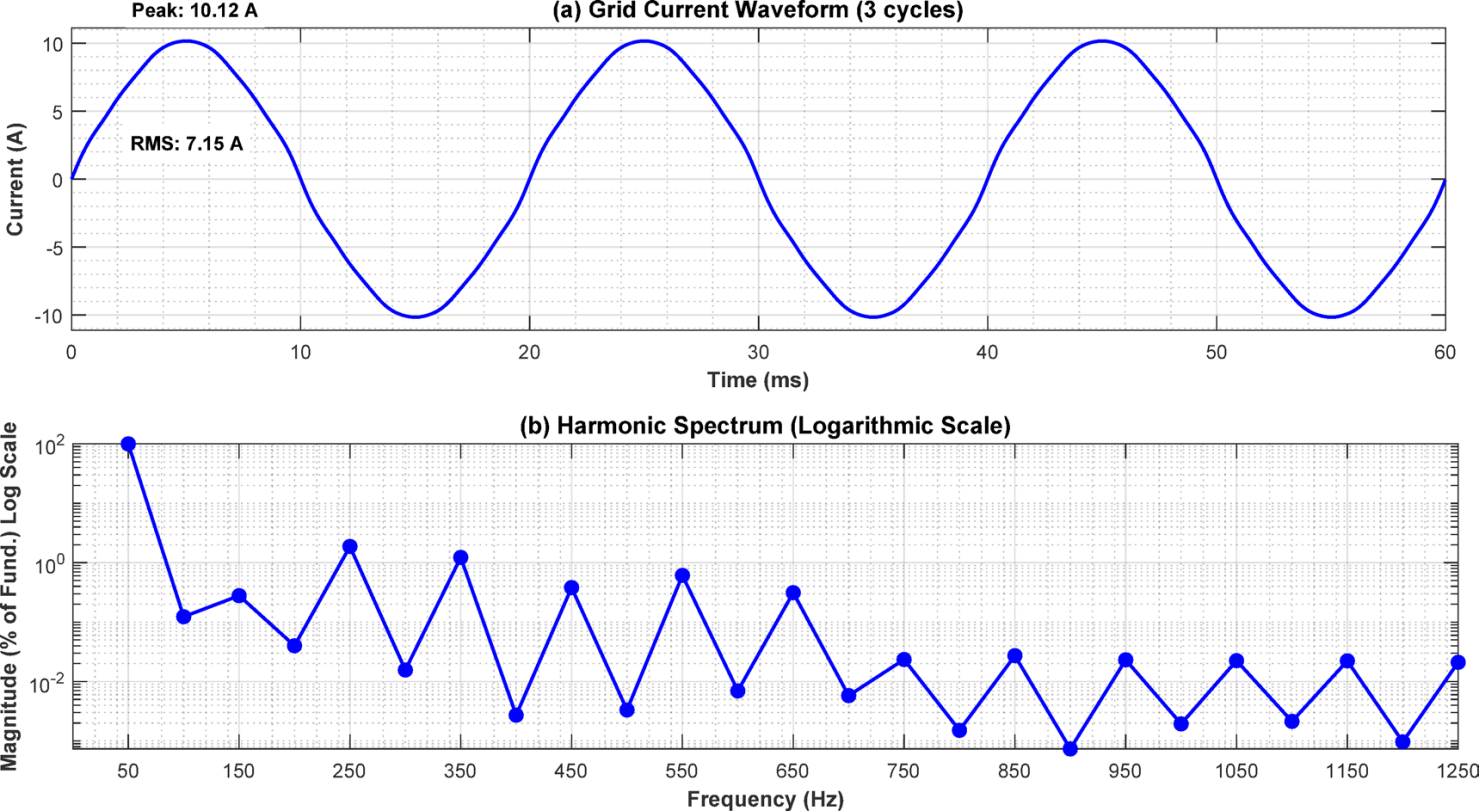
Grid current waveform and harmonic spectrum of the inverter output.

Figure 11 shows the grid current waveform and corresponding harmonic spectrum during grid-connected operation. The SPWM-based control, combined with the LCL filter and PLL-based synchronization, enables nearly unity power factor operation during grid-export mode. The overall control framework is compatible with modern interconnection requirements such as IEEE 1547 and IEC 61727 and can be extended to include detailed Low/High Voltage Ride Through and reactive power support functions in future hardware implementations.

### Explainability (XAI) metrics and validation

The proposed XAI-Fuzzy MPPT controller can be understood just as easily as each decision will be produced based on a transparent set of human readable IF–THEN rules. There are three popular explanation measures, which were considered: Fidelity, Consistency and Sparsity.

#### Fidelity score

Fidelity is the difference between the duty-cycle command represented by the fuzzy rules that were activated and the one that was actually generated. The controller with the same 20 Monte-Carlo scenarios used in MPPT performance assessment obtained a fidelity score of 0.96 and this means that the fuzzy rule base explanation of the internal decision-making process reconstructs it almost perfectly.

#### Consistency score

Consistency is a measure of how stable explanations are when small changes are made in the operating conditions. The calculated consistency score of 0.91 indicates that that the pattern of explanation is the same under noisy or fast-varying partial-shading conditions.

#### Sparsity index

Sparsity measures how far the controller can be reduced to a small physically significant number of features. This leads to a sparsity index of 0.38, which confirms that the controller relies on a small and easy-to-understand feature set (normalized power error E and its derivative ΔE), which does not impair control performance and increases transparency.

Bifaciality factor, battery capacity, and albedo, respectively, have the greatest contribution to the system efficiency (± 8.3 and 5.9 and 3.7, respectively) according to sensitivity analysis. The effect of temperature is rather small (± 2.1%). These findings suggest adaptive calibration is required prior to large-scale operation, especially of bifacial irradiance gains which are seasonally varying and site-dependent albedo conditions.

**Fig. 12:**
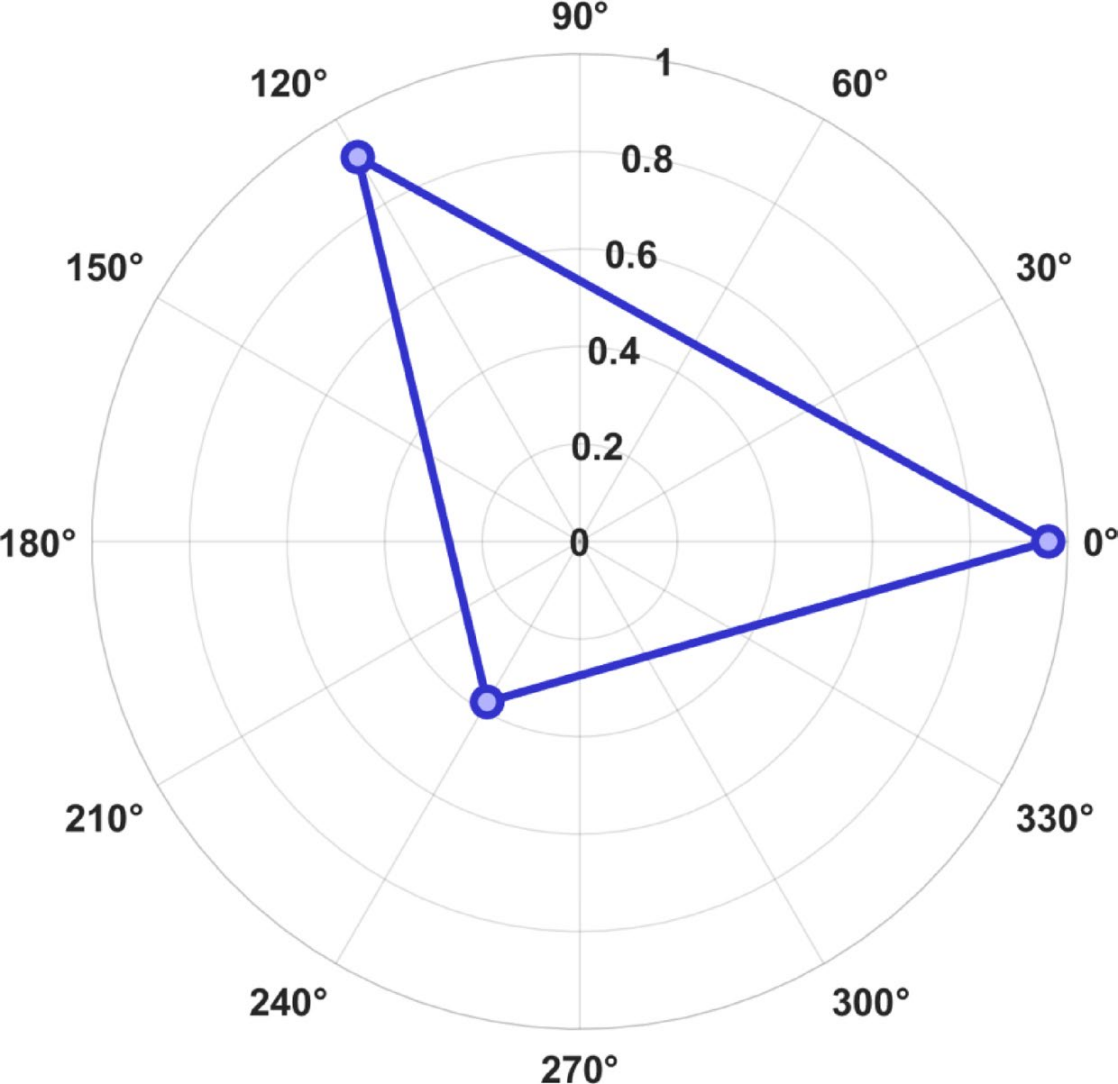
Spider plot showing explainability metrics: fidelity (0.96), consistency (0.91), and sparsity (0.38).

Figure 12 Spider plot showing XAI-Fuzzy controller in terms of explainability is high as shown by three main measures, namely, fidelity (0.96), consistency (0.91), and sparsity index (0.38), confirming transparent decision making. Unlike post-hoc methods to explain a model like LIME, SHAP or LRP, the proposed controller can be seen as highly interpretable as its decisions can be directly expressed in human readable IF–THEN statements. This natural transparency is especially appealing to safety–critical power system applications.

### Sensitivity analysis

Sensitivity analysis was conducted to learn the effect of the variation of some important parameters on the system performance. The parameters were adjusted by 20% with others being at nominal values, in terms of one-factor-at-a-time (OFAT) approach. Table 7 indicates the outcome obtained, which shows the parameters with the greatest influence on efficiency.

**Table 7:** Sensitivity analysis results.

Parameter	Nominal	− 20%	+ 20%	Impact (%)
Bifaciality (φ)	0.80	0.64	0.96	± 8.3%
Albedo (ρ)	0.25	0.20	0.30	± 3.7%
Temperature	25 °C	20 °C	30 °C	± 2.1%
Battery capacity	50 Ah	40 Ah	60 Ah	± 5.9%

The sensitivity analysis reveals that bifaciality factor has the largest impact (± 8.3%) on system efficiency, followed by battery capacity (± 5.9%) and albedo (± 3.7%). Temperature shows relatively modest impact (± 2.1%). These results indicate the need for adaptive calibration before large-scale deployment, particularly for bifacial irradiance gains which are sensitive to seasonal variations and site-specific albedo conditions.

## Conclusion and future scope

This paper achieves this by illustrating the design and operation of an XAI-capable adaptive fuzzy-logic-based MPPT and energy management system for a 10-kW bifacial PV-BESS-EV charging station at MMMUT, Gorakhpur, India. The offered hybrid system is effective in utilizing solar energy and providing stable, sustainable EV charging. The system enables explanably trained artificial intelligence, in addition to improved tracking precision, and thus represents the initial step toward trustworthy AI in the control of renewable energy.

The offered MPPT strategy provides a 4.5% increase in tracking efficiency (80.9%) compared to traditional P&O methods in partially shaded conditions, which is one of the most difficult operating conditions for conventional controllers. The hierarchical energy management system facilitates power distribution and enables renewable-based EV charging and self-consumption, aiming to provide grid support through smart battery scheduling. The system has good power quality, with a grid current THD of only 2.40%, well below the IEEE 519 limit. The explainability metrics are high (0.96), consistent (0.91), and low sparsity (0.38), indicating that the system’s decisions are accurate and interpretable.

Though there have been improvements, the proposed framework still has limitations. The current assessment is fully simulation-based; thus, hardware-in-the-loop (HIL) and field testing are necessary to confirm dynamic behaviour, converter nonlinearities, and real-world disturbances. Furthermore, reasoning with XAI incurs an additional computational cost, which can be challenging to run in real time on a low-power embedded controller. Lastly, bifacial gains are subject to seasonal variations and site-specific albedo conditions, meaning they will require adaptive calibration before large-scale implementation.

Research directions involve: (1) HIL testing and prototype development to test the design in the real world; (2) predictive control with machine learning (including LSTM models) to make the EMS more proactive and adaptive; (3) Vehicle-to-Grid (V2G) capability so that EVs can be active grid resources with the ability to do peak shaving and frequency support; (4) exploration of other XAI methods like symbolic regression or attention-based models to optimize efficiency and interpretation; (5) and economic and scalability: to evaluate the long- This research forms a very solid basis of the future generation of clean, smart, and grid-friendly EV chargers.

## Data Availability

The datasets generated and/or analysed during the current study are available from the corresponding author on reasonable request. Solar resource and atmospheric data were obtained from RETScreen Expert for the MMMUT Gorakhpur location (26.7° N, 83.4° E). Simulation models developed in MATLAB/Simulink R2023a can be made available upon reasonable request.
